# Contemporary specificities of labour in the health care sector: introductory notes for discussion

**DOI:** 10.1186/1478-4491-3-8

**Published:** 2005-08-18

**Authors:** Francisco Eduardo Campos, Eduardo da Motta e Albuquerque

**Affiliations:** 1 Center of Study of Collective Health, School of Medicine, Federal University of Minas Gerais (NESCON, UFMG), Minas Gerais, Brazil; 2 Center of Regional Development and Planning, Department of Economics, Federal University of Minas Gerais (CEDEPLAR-UFMG), Minas Gerais, Brazil

## Abstract

**Background:**

This paper combines the literature on public health, on economics of health and on economics of technological innovation to discuss the peculiarities of labour in the health care sector.

**Method and framework:**

The starting point is the investigation of the economic peculiarities of medical care.

**Results and discussions:**

This investigation leads to the identification of the prevalence of non-market forms of medical care in the countries of the Organisation for Economic Co-operation and Development (OECD). Furthermore, the health care system has a distinctive characteristic from other economic sectors: it is the intersection between social welfare and innovation systems. The relationship between technological innovation and cost in the health care sector is surveyed. Finally, the Brazilian case is discussed as an example of a developing country.

**Conclusion:**

The peculiarities of labour in the health care sector suggest the need to recognize the worth of sectoral labour and to cease to treat it separately. This process should take into account the rapid development of the health innovation system and one important consequence: the obsolescence of the acquired knowledge. One way to dignify labour is to implement continued education and training of health professions personnel.

## Background

Labour in the health care sector has specific characteristics that are evidenced by its institutional organization. The special economic properties of medical care determine the generalized emergence of what is called "market failures" in the economic literature. That is, the operation of market forces alone is not sufficient for the working of this sector, as is recognized in a recent report of the World Bank [1]. Society constructs varied institutional forms in order to offset market flaws by assigning an essential role to non-market institutions to render adequate services. The welfare state institutions may be viewed as an expression of social attempts to offset generalized market failures in the health care sector.

The next section of this paper presents the method of investigating the economic peculiarities of medical care as the starting point of this paper. The economic literature extensively discusses this subject, and the theoretical basis for social welfare institutions is well-grounded. This literature shows how superficial and theoretically poor are those approaches that wholly or predominantly insist on the market role for the operation of the health care sector. Kenneth Arrow's (Nobel economics prize, 1972) seminal contribution is a powerful remedy for such superficiality.

Arrow's analysis [2] is a conducting line for this paper. Arrow highlights that "the special economic problems of medical care can be explained as adaptations to the existence of uncertainty in the incidence of disease and in the efficacy of treatment". The weight of uncertainty and expressive information asymmetries determine the emergence of market failures, and hence the need of institutions to deal with such activities.

Based on these theoretical topics, the results of this approach are presented in the third section, which points to the prevalence of non-market forms of medical care organizations – an empirical confirmation of Arrow's analysis according to Barr [3]. Another specificity of labour in this sector is derived from this topic: the way these services are articulated (existing institutions, regulation and competition pressure) may affect both the quality of medical care and the pace of scientific research (and future scientific-technological progress as well).

The fourth section discusses a very distinctive characteristic of health care: its location in the intersection of two constituting institutional arrangements in advanced capitalist societies – the social welfare system is connected to the national innovation system. In other words, the professionals' performance in the sector affects – and is strongly affected by – the pace of scientific advancement and technological innovations. To simplify, one could say that a hospital is part of both systems [4].

In the fifth section, an assessment of this articulation between both systems introduces a discussion on cost peculiarities and those of technological progress in the health care sector.

The sixth section discusses the specific characteristics of the Brazilian situation. The precarious and rudimentary character of the social welfare institutions, the serious problems of access to health services and the general determinants of health conditions in Brazil, in addition to the incipient Brazilian innovation system (including that in health matters), simply add new problems to Arrow's list. Problems of resource allocation are crucial for defining the profile of the systems being settled, which amounts to one more labour peculiarity in the sector: that is, the involvement of professionals in the area in defining such a profile is not a trivial issue.

By articulating all questions so far developed, the seventh section concludes the paper by assessing the crucial role of recognizing the worth of labour in the health care sector and of ending its isolation.

## Method and framework

The starting point of this paper is the analysis of the economic peculiarities of medical care. Medical practice is full of lessons on the special character of medical care. Any physician or health care manager is able to describe a set of properties distinguishing the activity of a health professional from those of other workers in more conventional activities.

Perhaps one of the most often perceived differences in the sector is the lack of consumers' ability to choose their own basket of goods and services due to their lack of information for decision-making. It is useless to ask a patient whether he/she would prefer chemotherapy instead of radiotherapy in case he/she is able to afford only one of the alternatives. By the same token, it would also be useless to ask the patient whether he/she prefers an immunological examination or a magnetic resonance scan.

Such a situation is aggravated because the decision must be made at a time of personal or family distress – an illness threatens to take the patient's life or that of a loved one. For this reason, in contrast to other items whose consumption can be postponed, the consumer will make a heroic effort and will certainly not hesitate to try all available alternatives. This breaks one of the basic rules for adequate market resource allocation, as there is no symmetry of information. One side – the service provider – supposedly holds the information by accumulating esoteric knowledge [5] that is inaccessible to the other side.

Another important difference resides in the existence of limits to "rationalizing the production" as in other economic sectors. Every emergency service must always have a neurosurgeon available, even if traumas requiring nocturnal procedures rarely occur. It would be inadmissible to deny treatment to a person with multiple traumas on the basis of statistical evidence that his or her condition accounts for a small incidence of traumas and that it would not be economically justified to keep staff for this purpose. As a further example, even though snakebite is becoming increasingly rare, every health care unit must keep antivenin in stock – duly cooled and periodically checked – most of which is discarded.

Possible functions of standardized production are one more example of major differences. In industry, such possibilities stem from a relative standardization and monotonous industrial processes – the inputs are constant, the processes are repetitive and the outcome is always expected and predicted. In the health sector things are not as simple, as inputs and processes cannot be fully standardized.

An extensive literature shows that distinct health agents or even distinct medical "cultures" come to entirely different diagnoses regarding similar groups of patients submitted to them and prescribe utterly different therapies. According to the patient's social, economic and cultural level, the same disease may be given a totally different treatment. A verminosis, for example, may be treated with broad-spectrum drugs instead of a simple faeces examination or it could be treated using a series of examinations that could lead to a similar therapy (broad-spectrum drugs).

All this happens because there is much subjectivity in the health labour process, which remains basically artisanal, grounded on sight, touch and smell instead of being readily captured in a defined algorithm. Although a great deal of information may be objective – blood pressure, coronary permeability, electrocardiographic waves – other data or even the interpretation of those considered objective are quite subjective. There is always a feeling, a clinical look, based on subjective hints, that may trigger a dozen supplementary examinations. In addition, there is a sort of association between the physician and the patient that has been ontologically constructed during human history, ever since someone's sufferings were first alleviated by someone else.

Hindrances to standardization, however, should be qualified. In some health areas, there are – within certain limits – standard procedures, such as: laboratory procedures; highly standardized procedures in hospital sectors, resulting in serial surgeries; in public health, a number of advancements were achieved by means of standard protocols (e.g. for the treatment of diarrhoea and acute respiratory infections); medical procedures can be partially standardized through detailed classification (as for example, the Diagnosis Related Groups – DRGs).

Such elements, however, encounter a significant limit – the clinical contact controls all other procedures. And the clinical contact is based on much more shifting variables in which there are imponderables and from which it is difficult to construct closed algorithms.

Such observations constitute a source of empirical elements for a substantive theoretical elaboration of the peculiar characteristics of medical care as an economic category. Such peculiarities thus present a series of limitations to the market's ability to provide such services in a quantitatively and qualitatively adequate manner. Arrow had the merit to present this discussion based on an elaborate economic conception.

Arrow's paper [2] is pedagogically structured. Firstly, the market's working is described in accordance with the neoclassical economic theory that it should lead to the occurrence of a competitive equilibrium and optimum status. Then, the author poses hindrances to medical care marketability. The first basic difference from common commodities is related to the risk-bearing associated with medical care – "to a great extent a disease is an unpredictable phenomenon". A subtle outcome arises: "When there is uncertainty, information or knowledge becomes a commodity ..." But information, in the form of skilled care, is precisely what is being bought from most physicians and, indeed, from most professionals.

The elusive character of information as a commodity suggests that it departs considerably from the usual marketability assumptions about commodities." (p. 183). Thus, he sustains that "all the special features of this industry, in fact, stem from the prevalence of uncertainty". Finally, Arrow considers that "when the market fails to achieve an optimum state, society will, to some extent at least, recognize the gap, and non-market social institutions will arise attempting to bridge it" (p. 184). Therefore, such unique characteristics call for "a special place for medical care in economic analysis" (p. 186).

First, the demand for it is irregular and unpredictable (contrary to the demand for food and clothing, for example). Another important aspect is that the demand for health care is usually associated with an assault on personal integrity. A disease is not just a risk, but a risk associated with a cost per se (reduction or loss of labour capacity, even temporary, obviously affecting one's earning capacity), which is distinct from the specific cost of medical care (p. 187). Furthermore, there is an "opportunity cost": the time lost for earning in the labour market while undergoing treatment.

Second, the physician's behavior cannot be fully known in advance – medical care is one of the activities of which "the product and the activity are identical". In these cases, the commodity purchased cannot be tested before consuming and "there is an element of trust in the relation". The physician's behavior "is assumed to be ruled with concern to the patient's welfare, which is not expected from a salesperson". "In Talcott Parsons's terms, there is a 'collectivity-orientation', which distinguishes medicine and other professions from business, where self-interest on the part of participants is the accepted norm" (p. 187).

Other typical differences from business people would be that: advertisement and price competition are virtually absent among physicians; counseling by doctors concerning other treatment is supposedly given without self-interest; and treatment should be oriented by the needs of the specific case and not restrained by financial considerations (p. 187). Finally, resource allocation in this area is significantly affected by "ethic compulsion" (p. 188).

Third, there is uncertainty concerning the output – recovery from a disease is as unpredictable as its incidence: "Because medical knowledge is so complicated, the information possessed by the physician as to the consequences and possibilities of treatment is necessarily very much greater than that of the patient, or at least so it is believed by both parts. Further, both parts are aware of this informational inequality, and their relation is colored by this knowledge" (p. 190). Information asymmetry proves to have a crucial weight in the physician-patient relationship.

A relevant aspect should be added here: although the physician knows better than the patient, his/her knowledge is still extremely limited, given the extensive ignorance areas of scientific knowledge concerning the working of a human body, aetiology of a number of diseases, etc. Thus, there is a great difference between purchasing a chair from a cabinetmaker and a medical appointment – the cabinetmaker knows how to make the chair that the client desires, but the doctor has every chance of knowing very little about how to treat the patient or even being unable to do so.

Fourth, supply conditions are uncertain – entry into the market is not free, which restrains the assumption of full mobility of the production factors. Doctors must be licensed to provide medical services. Furthermore, medical education costs are high and apparently are only partially incurred by the student (p. 191), which means another dissociation from the requirements for the working of competitive markets, i.e. private benefits granted to students after graduation exceed private costs. Arrow associates the high costs of medical education with the quality requirements imposed by the American Medical Association (AMA) since the Flexner Report (p. 191–192) became known.

Fifth, pricing is uncertain – this topic is not usual in economic texts. There is an extensive price discrimination according to the patient's income, at one extreme reaching zero cost for indigent patients. Price competition is strongly disapproved.

Sixth, there exist indivisibilities – specialists and some sort of equipment constitute significant indivisibilities (p. 194).

As risk (of the disease and its treatment outcome) determines the medical care "market", Arrow calls for the possibility of an insurance market that might be able to organize and distribute such risks. If such a market were possible, the problems identified so far could be solved. However, the analysis of a hypothetical ideal insurance market (pp. 199–207) points to a set of problems:

• uncovered population segments (unemployed population, aged people, chronic disease sufferers, low-income population);

• differentiated risk pooling (if the market is competitive, high-risk individuals will tend to have to pay higher premiums);

• existence of moral hazard, to the extent that individuals covered by the insurance plans would tend to overuse them;

• adverse selection – an aspect pointed out by Akerlof [6], since in case premiums increased so as to cover elderly people, individuals bearing higher risks would be precisely those who would tend to agree to pay for the insurance (therefore, the individuals selected by the insurer would be exactly those with greater health problems: the costlier individuals for the system);

• uninsurable diseases (e.g. AIDS at the epidemic outset);

• existence of interdependent probabilities (when a problem affecting a person reaches other people, as in epidemics) [3];

• high administrative costs (which would serve as an argument in favor of quite generalized plans, particularly the compulsory ones).

Such problems determine the market's incapacity to provide a comprehensive insurance for medical care (p. 210). In a recent interview, Arrow [7] maintained the diagnosis of the 1963 paper, suggesting that funding the system through contributions to a centralized system "can be accomplished in a cheaper way than having many competitive insurance plans". An interesting aspect of this interview is that it confirms the basic elements of a diagnosis accomplished more than 20 years ago.

In the postscript to his original paper [2], Arrow highlights two aspects as follows: the failure of the market in developing policies of insurance against uncertainty has stimulated the emergence of many social institutions; in these institutions, the usual market premises are "contradicted to a certain extent". He notes that this is not an exclusive problem of the medical profession. All through the text, he emphasizes the role of nonprofit institutions in the sector (e.g. p. 191).

## Results: the prevalence of non-market forms of medical care in the OECD countries

Barr [3], in his evaluation of the social welfare states, points out that the structures of medical care organizations are internationally more divergent than those providing benefits, the other major function of the welfare state. According to Barr, there are various arrangements that can be grouped into three categories:

• a quasi-actuarial approach (purchase of private insurance by individuals and employees as well as private ownership of "medical factors of production" – as found in the United States);

• social security related to earnings (compulsory and funded by employees and/or employers' contributions sometimes supplemented by taxes, services rendered by a big private sector – Canada – or by a small private sector – Germany); universal medical services (funded by taxes and publicly owned and/or controlled factors of production – New Zealand, Sweden and United Kingdom);

• social service (most countries adopt schemes of this kind).

Another way of evaluating the distinct characteristics of medical care systems is accomplished by the OECD [8]. Its characterization does not contradict that explained by Barr (1992). In a different approach, Esping-Anderson [9] points to three categories of welfare systems: the Nordic, that of continental Europe and the Anglo-Saxon. This agenda contributes to a comparison between Arrow's diagnosis and the reality.

A general scenario can be drawn based on data from the World Bank Report [1], which show the relative weight of government expenditure in capitalist developed countries: 60% of total expenditure, according to the 1990 data. Even in the United States, where the private sector shows the highest participation among advanced countries, government health expenditure reached 6.17% of GDP in 2001, which amounted to 44.6% of total health expenditure (Table 1).

**Table 1:** Total health care expenditure, relative participation of private sector and public sector health expenditure

**Country**	**Total expenditure (% of GDP)**	**Private expenditure (% of total)**	**Public expenditure (% of GDP)**
United States	13.9	55.6	6.17
Canada	9.5	29.2	6.73
Sweden	8.7	14.8	7.41
United Kingdom	7.6	17.8	6.25
Germany	10.8	25.1	8.09
France	9.6	24.0	7.29
The Netherlands	8.9	36.7	5.63
Average: countries with high HDI	9.8	29.0	6.79
Brazil	7.6	58.4	3.16

Source: WHO (2001)

According to Barr's scheme, the American health system is one of those that is closer to the private market model: this system "shows those problems predicted in the theory". In terms of resource allocation, public expenditure covers exactly those areas in which policies designed for health insurance are not capable of paying for the risks: Medicare for aged people; Medicaid for the poor; military veterans (partially due to chronic health problems); maternity and children's welfare. Furthermore, an increasingly high cost and unequal access to services can also be found: at the end of the 1980s, 17.5% of the population above 65 years of age could not count on adequate insurance coverage in the United States [3]. Finally, based on an assessment of the most pro-market health care system, Arrow's view – outlined in his classical paper – is confirmed.

It is worth noting that the pattern of the American public expenditure on health is comparable to those in countries found at the other extreme of a description of welfare systems proposed by Barr: the Swedish government invests 6.8% of the country's total GDP in health (Table 1).

The issue concerning the effectiveness of the several institutional arrangements is very controversial. Hurst [10] compares the systems in the United States, Canada and the United Kingdom, concluding that the British system would be the most efficient, as it was the cheapest, with similar results.

An international comparison raises relevant questions on the relative effectiveness of health care systems. The World Bank report, for example, compares countries in terms of health expenditure and results [1]. According to this evaluation, the United States is at one extreme, i.e. the countries with the worst performance and highest expenditure. China is placed at the opposite extreme – better performance with lower expenditure (Figure 3.1, p. 54).

Such evaluation is not a trivial task. Measuring productivity is (usually) an old problem in economics and it is becoming even more serious with the emergence of information and communication technologies [11]. Measuring productivity in the service sector (where the health care sector is placed) is even more troublesome. Gordon [12] presents a general survey of the discussion for the United States. In assessing the growth rates of sectoral products by employee, he found that the health care services presented a positive variation rate only during the period 1960–1972. In the remaining periods (1972–1979, 1979–1987, 1987–1992), it was negative. According to Griliches [11], these services would be ranked among those hard-to-measure sectors (as opposed to measurable economic activities).

Although measuring productivity in the sector is troublesome and controversial, measuring cost increases is not; the difficulty here is to determine the reasons for the rise in medical care costs. What is consensual in the literature is the role that the structure of incentives plays in the cost dynamics and even in the direction technological progress will take in the sector [13]. In other words, the way medical care is organized (in the various existing structures) contributes to the definition of the activity performance and the expenditure policy.

In order to understand the effect of medical care organization on its performance, a study developed in Brazil is quite instructive. Campos [14] found that the physician's decision – considering all the degrees of liberty that professional autonomy grants him/her – strongly affects the consumption pattern and the impact of medical action on the epidemiological indicators. By studying the resolvability of health care services in homogeneous cities – whose differences are only those relative to the health professionals' labour ties – the author found a significant difference between a system hiring its professionals under a regime of labor exclusiveness as compared to a traditional system of multiple labour ties. An explanation for this would be that the first model forces an on-the-spot resolution of problems, since the lack of resolution of a problem implies the patient's return, sometimes with some inconvenience regarding time and circumstances to the professional.

The second model, by its segmentation, is limited to the traditional treatment prescription without taking account of the outcome of such a behavior, as a responsibility tie is not established between the professional and the patient. Campos [14] concludes that "working in an exclusiveness regime is the major determinant of such a differential behavior". In all dichotomies found in a tree of decisions studied – e.g. concerning the commitment to a drug prescription, laboratory examinations prescribed and hospitalizations, among others – significant differences in behaviour were found among medical personnel.

The outcome of this investigation can be generalized in terms of determining the way labour is organized considering the quality of its results. With regard to the United States, it is believed that the structure of medical insurance through third-party payments and fee-for-service encourages the overuse of services (leading to increasing prices). Barr [3] considers this structure to be one of the causes of high costs in the American system. The emergence of health maintenance organizations – HMOs – has been an alternative of shared responsibilities by the users and service providers, a way by which the agents share the consequences of increased expenses (cutting harmful incentives granted to third-party payments). The growth of HMOs is also related to encouragement of competition among service providers, a policy suggested by the World Bank to high-income countries [1].

Such changes affect academic research, however. Studies show that "in regions where managed care plans are dominant and where there is stiff competition for dollars and patients among hospitals, physicians at academic medical centers report more pressure to take care of patients – and thus conduct fewer human studies, do less clinical research, and publish fewer papers" (NSF) [15]. A problematic result of higher competition at the service level is that nowadays medical research is directly related to the future quality of medical care.

## Discussion: the health care sector's articulation of two institutional arrangements – social welfare systems and national innovation systems

The health care system possesses a distinctive characteristic relative to other economic sectors, i.e. it is the intersection between social welfare and innovation systems. These two systems (two institutional constructions) endeavour to surmount market restrictions. Arrow [16] points to a market economy trend to under-invest in research and development activities, which, as in the medical sector, would lead to the emergence of non-profit institutions so as to reach more desirable levels of R&D investment. These two institutional arrangements may be justified by Arrow's analysis [17], which considers that the market poses restrictions to efficiency (a task for the innovation systems) and equity (a task for social welfare systems).

The scientific and technological progress of nations – a decisive source of economic growth and development – is an outcome of complex institutional articulations involving firms, their R&D laboratories, universities and research institutions, financial systems, teaching institutions in general and the interaction of all such institutions, especially among firms [18,19].

The development of innovation systems is derived from a trend predicted by Marx [20], i.e. the systematic application of science to production. National systems of innovation may be studied as an institutionalization of this phenomenon discussed in the Grundrisse.

A national innovation system can be disaggregated into different sectors as the characteristics of technological progress vary significantly among the several sectors [21,22]. It is appropriate to say that innovation in the textile sector is quite different from innovation in the computer industry, i.e. the latter, for instance, depends much more on scientific knowledge and has a closer relation to universities and research outcomes [23]. Scholars of innovation economics have been surprised at the existence of a close relation between science and technology in the health care sector [24]. Following this rationale, the health sector can be outlined by the innovative dynamics differently from other economic sectors. Cautiously, however, the existence of an innovative subsector in the health care sector could be suggested.

A starting point already determined in the specific literature of the sector [25] is the notion of medical-industrial complex, which is an articulation involving medical care, education networks (schools, universities), the pharmaceutical industry and the medical equipment and diagnostic instrument industry. Back to that suggested formulation, the existence of an innovative subsystem within the health sector stemming from the literature on economics of technology and innovation adds to it a major aspect, i.e. it is necessary to study the information flows of technology and the mechanisms generating innovation in the medical-industrial complex. Gelijns & Rosenberg [26] presented a review of the literature on complex interactions between universities, industries and medical care systems, which pushed forward the advancement of medical technology. As in other sectors, interactions between demand for and supply of innovation are complex and varied.

On the one side, the study of a sectoral system necessarily contributes to the understanding of the medical care system. That is, the quantity and quality of treatment supplied, diagnostic methods and available equipment constitute a direct result of investment accomplished in scientific and technological research. By the way – and this is an important aspect for the specific objectives of this paper – a good deal of the "guilt" over the increased cost of the health care sector has been laid on technological innovation [3].

Lessons from the literature on economics of technology broke the traditional vision of technological progress, known as a "linear model". According to this model, there would be a process "from the top down", starting with basic research towards the laboratories of firms where applied research is accomplished and finally reaching the production phase. It could roughly be depicted in a scheme as follows: SCIENCE → TECHNOLOGY → PRODUCTION

This linear scheme is considered a distant representation of reality. Sources of technical progress are much more complex. For example, solving problems and bottlenecks in production is a major source of innovation (this is the way new methods of production appear). In many instances, the arrows' sense is the opposite – radio astronomy has been developed as a new scientific discipline based on the work of two physicists (Penzias and Wilson), employees at the laboratories of the Bell Company, in an attempt to solve a noise problem in transcontinental telephone calls. Therefore, science can be viewed as both leading and following technological advances [19]. For this reason, the existence of a dynamic nucleus of firms is crucial for the maturing of national innovation systems.

Hospitals play a major role as an authentic reservoir of innovation "coming from the top". Hospitals contribute to scientific development, i.e. the arrows of the scheme above point to both sides in the health sector, too. As a matter of fact, service-providing would usually play a similar role to that of firms in other sectoral systems: solving problems and escaping from bottlenecks is a significant source of innovation [4].

What is peculiar in the interaction between health innovation systems and health care systems is their closer tie to each other and the more immediate impact between technological progress and social welfare, the latter a decisive component of economic growth sources. Therefore, innovation in the health sector would have a double effect on the economic dynamics in general – the "usual" effects of every innovation and also those on health and welfare.

The relevance of such an articulation simply adds a new peculiarity and a new source of labour heterogeneity to the sector. It would not be possible to grasp the health care system integrally by neglecting academic research in the life sciences. The life sciences spent 54.4% (USD 10.83 billion) of total resources for R&D in academic institutions in 1993. In that year, the total United States expenditure on R&D amounted to USD 134.4 billion [27]. In 1994, the health sector absorbed 16.5% of federal expenditure on R&D, which reached USD 68.33 billion. The weight of R&D investment in health can also be assessed in the industrial sector. Drugs and medicines were the industrial sector with the most intense R&D (R&D expenditure in relation to revenues), and the sector of optical and surgical instruments and others was placed fifth [27]. Total expenditure on biomedical R&D funding reached USD 30 billion in 1993 [28]. Industry accounted for 50% of the total. American industry increasingly depends on the funding of science with public resources; the leading position of the biomedical sector is highlighted in this regard [29].

Therefore, it is possible to trace a labour repositioning, in relation to which the pole constituted by activities connected with intellectual labour can be found [30]. The peculiarity of labour repositioning in the health care sector would not reside in the shift of functions of manual labour (as in the industrial sector), but in the increased participation of more skilled professionals (including scientists and researchers), in addition to the demand for better-trained professionals in the area to deal with diagnostic methods and electronic equipment, etc. Such a dynamic stresses the need for training and retraining the whole set of professionals in the sector: the speed of technological progress enhances the role of continued learning.

## Discussion: technological innovation and cost in the health care sector

The technological innovation dynamics in health care has been considered one of the reasons for the increased expenses in the sector. This would be partly explained by another peculiarity in the sector, i.e. the cumulative introduction of technology, as opposed to other production sectors, where new technology replaces the old. For example, the introduction of cardiac imaging (electrocardiogram, echocardiography, Doppler) did not replace traditional auscultation; the old technology and the new are used interchangeably. The obstetrician works with the time-tested Pinnard stethoscope together with modern sonar devices in order to listen to the fetal heartbeat.

In the case of the United States, there is another question derived from the pressure the demand for medical care exerts on R&D activities: the health insurance mode of organization based on retrospective payments (the mode of medical care and the incentive structure hence derived) exerts pressure on R&D activities in terms of producing costly and effort-consuming innovation [13]. Recent changes in the system would lead to a reversal of this pressure so as not to encourage the use of expensive technologies. As an example, General Electric had frozen the development of a diagnostic technology called positron emission tomography (PET) which "produces tridimensional images reflecting chemical and metabolic activities in the tissues". The reason for such a freeze, according to the company, was that government was too stringent in approving reimbursement to patients for PET scans. Previously, the company had invested heavily in developing computerized tomography scanners (CT) and magnetic resonance imaging (MCI), and had taken them to the market [13].

Such sensitivity in terms of technological progress in relation to the incentive structure is quite important. Two examples of how the structure of service provision affects both its quality [14] and the degree of involvement of hospital units with research [15] were presented in the third section. Surveying this subject, Halm & Gelijns [31] say: "it is clear that the critical point here is not medical technology per se, but a combination of economic, professional, and social incentives in the health care system which tend to diminish apprehensions as to the decision-making in medical care".

There is evidence that technological innovations are not exclusively price-raising in medical care [13]. This is an open question in the OECD document, which is uncertain "whether new technologies are part of the problem, part of the solution or both" [32].

In order to analyse this aspect, Weisbrod compares vaccines and transplants, their costs, effects and respective demands for innovation. He used the biologist Lewis Thomas' elaboration, which distinguishes three technological development stages in medicine so as to make his position explicit:

• at the lowest level, the non-technology level, where the relation between the patient and the disease is entirely understood. Little can be done by the patient, hospitalization and infirmary services, and there is little hope of recovery (untreatable cancer, severe rheumatoid arthritis, multiple sclerosis, advanced cirrhosis);

• at a somewhat higher level, halfway technologies, which would include dealing with the disease and its disabling effects. These are technologies that adjust the patient to his/her disease and postpone death (artificial organ implants, cancer treatment by means of surgery, radiation and chemotherapy);

• high technologies, as for example immunization, antibiotics and prevention of nutritional disorders, are designed for diseases whose mechanisms are known and whose treatment/prevention is feasible.

Weisbrod suggests the use of the following dynamic scheme: historically, knowledge goes from the first kind of technology to the second and then to the third. From this scheme, it arises that the cost function associated with such a dynamic process is an inverted U-shaped one. In the case of non-technology, not much can be done and the costs are low. The most expensive would be that of intermediate technologies, then decreasing again in the third stage – high technology. Weisbrod uses the evolution of poliomyelitis as an example: in the beginning (two generations ago), its victims died rapidly as a result of paralysis; then the development of intermediate technology came about with the emergence of the iron lung, which prolonged the patient's life at high cost; finally, the vaccines (Sabin and Salk) in the high technology phase reduced dramatically the costs associated with polio [13].

Based on this scheme, Weisbrod suggests, for the case of the United States, that "the development of halfway technologies was implicitly encouraged by the cost-reimbursement insurance system that has dominated hospital and medical care until recently, because there was little or no incentive for medical care providers to avoid costly technologies that were even marginally effective". (p. 534). That is, it is not the technology that accounts for increased costs, but the scheme of incentives that guides its evolution.

Further on, Weisbrod lists the technologies with a demand for health insurance: "the demand for health insurance tends to increase most rapidly when changes in technology are of the expenditure-increasing, halfway type" However, high technologies (vaccines) would tend to diminish the demand for insurance."

In order to confirm his suppositions, he describes some impacts of the emergence of HMOs being more concerned about costs; they would have broadened their R&D profitability by directing their actions to: drugs that could avoid costly treatments; drugs that replace surgeries (e.g. cimetidine, a substitute for ulcer surgery) [13]. Lichtenberg studied the relation between new medicine and the demand for hospitalization and found that hospital bed-days declined rapidly "for those diagnoses with a larger number of drugs prescribed and a greater change in distribution of medicines". He estimated that an increment of 100 prescriptions is associated with a reduction of 16.3 days of hospitalization [33].

However, perhaps Weisbrod has added another problem to Arrow's paper. As it is basically bought to pay for hospital treatments, health insurance provides incentives for R&D to search for ways to treat patients rather than prevent diseases. And this is not "optimum" in social terms.

Weisbrod's analysis is interesting as it contributes to assessing the demand for innovation in the health sector and mainly to show how the sector's organization affects technological progress. However, in the debate on health care changes, only the side of the demand for technological innovation has been focused upon; the conditions governing the supply of innovation have been neglected [26]. Undoubtedly, further developments in cancer prevention are restrained in part by the state of science.

A good example of such a restraint is provided by biotechnology: "a revolution in health care", announced OCDE [32]. The development of genetic therapies might mean treating cancers, genetic diseases and others (such as rheumatoid arthritis). Some research efforts are in the phase of clinical trials. However, the development of such therapies is complex and difficult, and so far "clinical efficacy has not been demonstrated in any of the genetic therapies". Perhaps "the genetic therapy will take a long time before reaching the patients" [32].

However, it is possible to conjecture that the biotechnology revolution will make high technology-type innovation available as soon as it is developed and employed in health care systems, in accordance with Weisbrod's scheme – efficacy derived from the understanding of the processes of a number of diseases treated with (cost-reducing) vaccine-type therapies.

## Discussion: issues concerning the situation in Brazil

So far the discussion has centred on advanced countries. A brief introduction to the situation in Brazil requires caution so that conspicuous differences may not be neglected.

The first major difference is the country's development stage. According to the World Bank, Brazil is a high-medium income country, with a GDP per capita of USD 3640 in 1995, and was placed 46th in the world ranking [34]. In relation to the Human Development Index (HDI), it was ranked 58th in 1993 and 62nd in 1995. The technological gap and the social gap come together. If expressed by the terms used all through this paper, such a reality can be translated into precariousness of social welfare systems in the country (with severe influence on its health care structure) and the rudimentary and incipient character of the national innovation system [35].

This standpoint contributes to determining the scenario of health and morbidity in the country. Brazil has been undergoing, according to the jargon used in the health milieu, an epidemiological transition, which combines features found in a low-income country, such as sanitation shortages, malnutrition and infectious-parasitic diseases, with features of high-income countries, such as a growing incidence of degenerative diseases. Such differences pose complex tasks to be tackled by the whole health apparatus in the country.

As for the labour peculiarity, the health care sector must count on broad competences, ranging from the treatment of simple verminous diseases to modern techniques of emergency treatment. A neurosurgeon and a plastic surgeon are not luxuries – these two kinds of specialists are needed in a country with a high incidence of labour accidents such as Brazil (one need consider only the kind of accidents found in construction and those accidents requiring hand-repairing surgery, recovery of persons with burns, etc.).

To an extent, the capacities the advanced countries have been building through time – which have been substituting for each other since their introduction – must coexist in the country. The result is a health care system more complex and differentiated than those of countries at either extreme (high-income countries: no verminous diseases and better labour conditions; low-income countries: lower incidence of degenerative diseases).

Another effect of Brazil's economic stage is the existing budgetary restriction: basic necessities (education, sanitation, health, infrastructure investment) compete with each other in budgets with relatively scarce resources.

The World Bank proposal for governmental action is based on an intervention divided into three basic levels, corresponding to three different foundations [1]: alleviation of poverty and access of the poor to health care services; public health; extension of medical care to the population through insurance as well as its regulation. The World Bank proposal concentrated the discussion on the latter (insurance and regulation). The Brazilian peculiarity would be the relevance of an action based on a combination of these three levels [1].

That the Brazilian constitutional text has adopted the proposed organization of a single health care system (SUS) that is universal and equitable, with an integral and socially controlled approach, is equally a significant conceptual advancement and a great operational complication, when the health care scenario in the country is considered.

Boelen [36] proposes a comparative analysis of the social accountability of health care services that are oriented by four polar concepts: equity versus quality and relevance versus cost-effectiveness. According to the author, it would be relatively easy to design an equitable system by following a formula containing only the basic health care actions for the most vulnerable population groups. In the same way, it would be theoretically simple to construct systems following the quality criterion alone, unconcerned with the coverage of the actions developed. In this case, the concept of relevance of the health care actions would oppose the analysis of their cost effectiveness.

When writing its constitution chapter on health, and hence including the SUS proposal, Brazil posed a great challenge to itself, i.e. to reach the four cardinal points (equity, quality, relevance and cost-effectiveness) at the same time. The very fact that the health chapter of the Brazilian constitution is within the social security area, joining health care actions with social security and social work activities, accounts for the size of such a challenge.

The difficulty resides in correctly developing the matching of such elements. As compared with more advanced countries (Table 1), it is worth assessing the need for and possibility of a general increase in health care expenditure (public and private). Public expenditure, by strengthening basic programmes, public health and investment in regulatory activities (the recent scandal of falsified drugs is tragic proof of the price to be paid for weakness in such areas) cannot be replaced.

Based on the ideas discussed throughout the present paper – that the health care system is placed in the intersection of the welfare system and the innovation system – the relevance of social and economic investment in research should be considered. Investment should be made in the country to enhance scientific and technological capacity in biotechnology as well as to improve and extend sewage systems. The range of activities to be achieved by the construction of these two indispensable systems is sizable.

From the viewpoint of technological innovation in health care and that of the rest of the innovation system as well, the technological gap of the country, in relation to the international technological frontier, reveals some advantages and requires some efforts [37]. The advantages are as follows: investment in initial phases of development is not necessary, as the country is in its absorption phase of technologies generated in the technological frontier; it is quite possible for the country to adopt a technology after defining its development "path" (i.e. expenditure on technologies that could later be replaced by new developments can be avoided).

However, such advantages cannot be exploited without making important domestic investments – constructing "absorption capacity" is a must, since: the absorption and necessary adaptation of such technologies are not passive processes; they require knowledge, critical mass, financial and entrepreneurial capacity; they presuppose a follow-up and monitoring capacity of current scientific and technological progress, to the extent that basic research frequently means an entrance ticket to a circuit of scientific and technological information, as pointed out by Mowery & Rosenberg [38]; previous knowledge is necessary even for a simple purchase of equipment, machines and processes.

## Conclusions: recognizing the worth of sectoral labour and integrating it

The incapacity of the market to allocate resources in health care production, the asymmetry of knowledge and the relation of trust between the physician and the patient are among the reasons why attempts at external regulation and control of health care labour have failed or can be bypassed.

The traditional compendiums of health administration have already recognized that the three basic modes of health care labour remuneration – fixed-salary payment based on time spent in the procedure, fee for service and the different modes of capitation – present advantages together with remarkable failures as to its performance controllability.

Those receiving fixed salaries tend to evade providing services, unlike those paid for procedures accomplished, who tend to overestimate service provision, while mechanisms for capitation may be bypassed through selecting less vulnerable groups. Perhaps for these very reasons, these mechanisms are rarely used separately, and there is a trend towards combining them, as for example, incentives for increased productivity combined with wages.

No matter how creative health service managers may be, there will always be circumstances standing in their way, aggravated by a disguised protection of some attitudes by corporatism, professional complicity or a strictly organized hierarchical structure. There are differential requirements that are rarely unequivocally expressed beforehand. A clear example of this is the strict control of the hours worked by subordinates, on the one hand, and a relative leniency with physicians as to the same requirement, on the other hand. Another striking example of such a situation is the failure of management to attempt to limit the number of examinations and hospitalizations provoked by a given number of visits to the doctor.

First, standardization is impossible if the input of this system is not known, i.e. the seriousness and complexity of the pathologies to be treated. Furthermore, the control of the denominator of this equation is very difficult, as it consists of visits and not of assisted patients. As it would hardly be reasonable to forbid the patient's return visits, which would be desirable as a demonstration by the service provider of concern with solving the problem, the number of visits for a similar group could be unnecessarily multiplied, which would allow a striking inflation of procedures, in this way bypassing the external control.

For all these reasons, a tight wage policy and precarious health care labour conditions, instead of saving resources, may result in their waste by provoking an increment of unnecessary examinations and hospitalizations that could be avoided if a specific agreement to solve the problems were reached. In this case, a positive labour incentive – including improved wages and working conditions and encouragement for training, i.e. positive organizational "climate" and "culture" – would certainly represent a positive impact on health conditions without a burst of final costs.

In the Brazilian experience it can be said that, on the negative side, doubling the salaries of social security physicians after months of strike in the early 1990s did not have measurable positive impacts on productivity and outcome, despite the economic burden for the public accounts. In contrast, some managerial micro-decisions with low added costs – such as personal recognition, prestige, kindness in interpersonal relations and a smooth work environment, encouragement to participate in scientific events, flexibility in order to attain personal expectations and demands – can have positive impacts in the outcomes. Therefore, it seems clear that it would not be possible to rationalize health care labour without the workers' adherence and collaboration, in an agreement that could simultaneously benefit users and providers.

Additionally, viewing the worth of labour exclusively as a wage issue would be simplistic, although it is still crucial. Another way to dignify labour is by implementing continued education and training of professional staff. This process should take into account the rapid development of the innovation system whose consequence is the obsolescence of acquired knowledge. More than half of medical techniques are estimated to become useless in 16 to 18 years. The contribution of the academic apparatus – which was so relevant during the medical education "boom" period about 20 years ago and surely made this professional activity available to large population contingents, previously unassisted – will be lessened if such institutions continue giving only initial education background to physicians and other professionals in the sector, which was a very important mission when innovation was a relatively slow-paced process.

## Competing interests

The author(s) declared that they have no competing interest.

## Authors' contributions

FEC and EMA shared the research and co-authored this paper.
